# Short Stature and Celiac Disease in Children (5 to 16 Years) Presenting at a Tertiary Care Hospital in Peshawar

**DOI:** 10.7759/cureus.26099

**Published:** 2022-06-19

**Authors:** Amir Muhammad, Nauman Arif, Khawaja Kamran Wajid, Khalid Rehman, Naila Sardar, Palwasha Khan, Umar Hussain

**Affiliations:** 1 Pediatrics, Lady Reading Hospital, Medical Teaching Institute (MTI), Peshawar, PAK; 2 Epidemiology, Khyber Medical University, Peshawar, PAK; 3 Public Health, Khyber Medical University, Peshawar, PAK; 4 Pediatric Medicine, Lady Reading Hospital, Medical Teaching Institute (MTI), Peshawar, PAK; 5 Orthodontics, Saidu Medical College, Swat, PAK

**Keywords:** gluten, enteropathy, children, short stature, celiac disease

## Abstract

Background: Malabsorption is the typical presentation of celiac disease in early childhood, whereas older children can present with extra-intestinal symptoms including short stature and delay in pubertal development.

Objective: To determine the frequency of celiac disease in shortening of stature in children.

Material and methods: This descriptive, cross-sectional study was conducted at the Pediatric Department in Lady Reading Hospital, Peshawar, on 152 short stature children of both genders aged 5 to 16 years. Children with dysmorphic faces, syndromes, endocrine disorders, and children or their parents who refused to give consent were excluded. Anthropometric measurement was done on standard equipment. Height and weight were plotted on WHO centile charts. All the children fulfilling inclusion criteria were advised serologic anti-tissue transglutaminase antibodies tests.

Results: Overall the frequency of celiac disease was 33.77% (n=51) among the children with short stature. The mean age of the study was 6.71±1.52 years. There were 76 males (50.3%), while there were 75 females 49.7%. The frequency of celiac disease among short stature females was higher (n=28, 54.9%) than short stature males (n=23, 45.1%). However, the results were not statistically significant (P=0.358). The frequency of celiac disease stratified by age group was not statistically significant (P=0.491).

Conclusion: One-third of children having short stature have celiac disease. The frequency of celiac disease in children with short stature has no association with gender and age.

## Introduction

Celiac disease, also known as gluten enteropathy, causes damage to the intestinal mucosa by an autoimmune reaction leading to villous atrophy. Villous atrophy then leads to multiple micro and macronutrient deficiencies [1,2]. Worldwide its prevalence is about 1% affecting females twice more than males [3]. Malabsorption is the typical presentation in early childhood, whereas older children with celiac disease can present with extra-intestinal symptoms including short stature and delay in pubertal development. Exclusion of gluten from diet generally results in growth catch-up and normal pituitary function [4]. According to the European and North American societies guideline, celiac disease is diagnosed on the basis of serological tests and small bowel biopsy histological examinations [5,6].

By definition, short stature is height less than or equal to 2 standard deviations below the mean for the age and sex or height below 3rd centile [7,8]. short stature can be the result of normal growth variants e.g. familial or constitutional or it can be the result of chronic systemic disorders, endocrine disorders, genetic, chromosomal disorders, and skeletal dysplasia. Chronic malnutrition and chemotherapy can also result in short stature [9].

Singh et al. [9], showed an 11% prevalence of celiac disease among children presenting with short stature at a tertiary care hospital. Results from two European studies on short stature show celiac disease prevalence at 2:180 and 0:149 respectively. In short stature children without gastrointestinal signs, the prevalence of celiac disease raised up to 2-8%. By excluding endocrine causes of short stature, celiac disease prevalence will raise to even 59% [10,11]. 

The rationale of this study is that both celiac disease and short stature are common reasons for pediatric patients presenting to the pediatric department in tertiary care hospitals, but the prevalence of celiac disease in short stature children is not known in our country. Previous studies have shown an increased frequency of celiac disease in short stature children than normal. But to our knowledge, no proper studies have been conducted to know the role of celiac disease in the etiology of short stature. Celiac disease is a treatable condition, so by an early diagnosis, we can treat the short stature at the proper time. The objective of this study was to determine the frequency of celiac disease in the shortening of the stature of children.

## Materials and methods

This descriptive cross-sectional study was conducted at the Pediatric Department, Tertiary Care Hospital, Peshawar, Pakistan, from 1st July 2021 to 30th October 2021. Lady Reading Hospital Ethical Board Peshawar, Pakistan issued approval (627/LRH/MTI) for the present study. By using open epi software (www.openepi.com ), the total sample size was calculated to be n=152 at 95% confidence interval, 5% margin of error, and 11% frequency of celiac disease in short stature, based on a previous study [9]. Sample selection was done by using the nonprobability consecutive technique. Children of both gender aged 5 to 16 years old, meeting criteria of short stature were included. Children with dysmorphic faces, syndromes, endocrine disorders, and children or their parents refusing to give consent were excluded.

Celiac disease in children was confirmed with positive serologic tests with anti-tissue transglutaminase antibodies more than 10 times upper normal. While short stature was defined as children with height ≤ 2 standard deviations below the mean for the age and gender or height less than 3rd centile.

The demographics were recorded like age, gender, parental education, and family type. Weight was recorded for each child using a standard balance. Height was measured in meters with a measuring tape. All the children fulfilling inclusion criteria were advised serologic anti-tissue transglutaminase antibodies tests (Anti TTG IgA and Anti TTG IgB). The variables involved in our study are pre-validated in the literature [12]. Bias and confounders in the study were controlled by strictly following inclusion/exclusion criteria and stratification.

Data collected were analyzed using R Package version 4.1.2 (www.r-project.org). Quantitative variables like age were expressed as mean and standard deviation. Qualitative variables like celiac disease and gender were expressed as frequency and percentages. A Chi-square test was applied to determine the association of celiac disease with gender, age group, parental education, and family type. Logistic regression was applied for the significant variable using celiac disease as the dependent variable. A p-value of ≤0.05 was considered statistically significant.

## Results

Overall the frequency of celiac disease was 33.77% (n=51) among the children with short stature. There were 50.3% males (n=76) and 49.7% females (n=76). The most common age group was 6.1 to 8 years (n=61, 40.4%) followed by 4.5 to 6 years (37.7%). Most of fathers were literate (n=120, 78.94%) followed by illiterate (n=32, 21.05%). Similarly, most of mothers were literate (n=105, 69.07%) followed by illiterate (n=47, 30.92%). Most common family type was joint (n=98, 64.5%). The mean age of the study was 6.71±1.52 years while the mean weight and height were 14.19±2.48KG and 103.06±7.31 cm respectively (Table 1).

**Table 1:** Frequency of gender, celiac disease, age group, parental education, and family type of the participants

Variable	Characteristics	n(%)
Gender	Male	76(50.3)
	Female	75(49.7)
Celiac disease	Yes	51(33.77)
	No	100(66.23)
Age group (years)	4.5-6	57(37.7)
	6.1-8	61(40.4)
	above 8	33(21.9)
Father's education	Literate	120 (79)
	Illiterate	32 (21)
Mother's education	Literate	105 (69)
	Illiterate	47 (31)
Family type	Joint	98(64.5)
	Nuclear	54(35.5)
Age (years)	Mean ± SD	6.71±1.52
Weight (Kg)		14.19±2.48
Height (cm)		103.06±7.31

The frequency of celiac disease among short stature females was higher (n=28, 54.9%) than short stature males (n=23, 45.1%). However, the results were not statistically significant (P=0.358). The frequency of celiac disease stratified by age group was not statistically significant (P=0.491). The highest frequency of celiac disease was for the age group 6.1-8 years (n=24, 39.35%). A very highly statistically significant association was found between paternal education and celiac disease (P<0.001). Celiac disease was more prevalent in cases whose father were illiterate (n=31, 96.9%) than literate (n=21, 17.5%), statistically (P<0.001). In a similar way, a very highly statistically significant association was found between maternal education and celiac disease (P<0.001). Celiac disease was more prevalent in cases whose mothers were illiterate (n=45, 86.5%) and of primary level (n=6, 11.5%). The association between family type and celiac disease was not statistically significant (P=0.851) (Table 2).

**Table 2:** Frequency of celiac disease stratified by gender, age, parental education, family type *Fisher exact test/Chi-square test

Variable	Characteristics	Celiac disease		P-Value
		Yes	No	
Gender	Male	53(53)	23(45.1)	.358
	Female	47(47)	28(54.9)	
Age group (years)	4.5-6	40(70.2)	17(29.8)	0.491
	6.1-8	37(60.7)	24(39.3)	
	above 8	23(69.7)	10(30.3)	
Father Education	Literate	99(88.2)	21(17.5)	<0.001
	Illiterate	1(1.3)(8)	31(96.9)	
Mother Education	Literate	98(93.3)	7(6.7)	<0.001
	Illiterate	2(4.3)	45(95.7)	
Family type	Joint	65(65)	33(63.5)	0.851
	Nuclear	35(35)	19(36.5)	

The odds of celiac disease in children whose fathers were illiterate were 44.44 times more than in literate (P=0.002) statistically. Similarly, the odds of celiac disease in children whose mothers were illiterate were 164.97 times more than in literate (P<0.001) statistically (Table 3).

**Table 3:** Association of celiac disease with parent’s education *OR; Odds ratio, logistic regression

Exposure		Celiac Disease		Univariate			Multivariate		
		No	Yes	OR	95% CI	p-value	OR	95% CI	p-value
Father education	Literate	99 (82.5)	21 (17.5)	-	28.88-2673.99	<0.001	-	4.78-1011.33	0.002
	Illiterate	1 (3.1)	31 (96.9)	146.14			44.44		
Mother education	Literate	98 (93.3)	7 (6.7)	-	77.26-2209.71	<0.001	-	36.52-1244.03	<0.001
	Illiterate	2 (4.3)	45 (95.7)	315			164.97		

## Discussion

This study aimed to determine the frequency of celiac disease (CD) among children presenting to a tertiary care hospital. Our main findings showed that overall the frequency of CD was 33.77 among the children with short stature. 

Short stature is defined as children with a height ≤ 2 standard deviations below the mean for the age and gender or height less than the 3rd centile. Celiac disease was defined as children with positive serologic tests (anti-TTG IgA titer more than 10 times the upper limit of normal. A similar methodology was applied in previous literature [12,13].

We used only serological tests to diagnose CD. The recent update in the diagnosis of CD on the basis of serology alone may further enhance the evaluation of patients with short stature [14]. Per the recent European Society for Paediatric Gastroenterology, Hepatology and Nutrition guidelines, a patient with anti-tTG-ab levels ≥ 10 times the upper limit of normal along with positive anti-EMA on a second blood draw can be diagnosed with CD. This suggests that CD can be diagnosed without duodenal biopsy in a subset of patients with short stature. However, it is important to note that in patients not meeting the abovementioned criteria, a duodenal biopsy should be performed to confirm the diagnosis. Also, a duodenal biopsy may improve the yield of diagnosis in patients with high clinical suspicion like those with short stature and concomitant anemia or chronic diarrhea or with idiopathic short stature even when the serological tests are negative.

The mean age of the study was 6.71±1.52 years. Approximately similar mean age (8.368 ± 2.387 years) was reported in a previous study conducted in Faisalabad on the prevalence of CD in idiopathic short stature [12]. Another study conducted in Iran also reported similar mean age [13]. Our findings showed that there were 50.3% males, while there were 49.7% females. This shows that short stature equally affects both genders in our population. But a previous study conducted in Pakistan shows female predominance [12].

Our results showed that overall the frequency of CD was 33.77% (n=51) among the children with short stature. This shows that every third participant having short stature is likely to have CD. It has been reported that 45 % of celiac patients have short stature [13]. Another study conducted in Iran by Hashemi et al. on 104 children reported that the prevalence of CD among short stature children was 33.6% [14]. These results are consistent with our findings.

Other studies showed a variable prevalence of CD among short stature children and range from 18 to 59 [15,16]. Why growth retardation occurs in individuals affected with CD is clearly understood but deficiency of nutrition like Zinc, low level of somatomedin in the serum, and low growth hormone can have a role [17,18].

Recently a meta-analysis was published in 2020 on the prevalence of celiac disease in patients with short stature. They included 17 studies in their analysis [19]. Their results showed that three studies reported sero-prevalance of CD among short stature children and it was 11.2 (95% CI=4.5 -21.2) [20-22]. These results are different from our study. The difference can be due to genetic factors. Results vary in different populations, hence, the CD shows genetic susceptibility.

Another study conducted in Saudi Arabia on 275 retrospective cases found that the prevalence of CD among short stature children was 5.8 [23]. And another study was conducted in the Indian population on 1000 children with short stature having an age range of 5 to 10 years. They reported that CD is very less common (1.9%) among short-stature children [24].

Our results showed a positive association between parental educational level and celiac disease. Celiac disease was more common in children whose parents were either illiterate or had a primary level of education. To the best of our knowledge, no such association has been reported in the literature.

This is the first of its kind study on the frequency of CD among short stature children in Peshawar in a major tertiary care hospital. Most of the patients of the Peshawar population are presenting to this hospital. So this study can show relatively good statistics.

There are a few limitations to this study. First is the weak design which may not show the real association of CD with short stature. A case-control study can depict association in a far better way. A community-based and case-control study can show the real association between CD and short stature. The gold standard for diagnosing CD is the jejunal biopsy, and we used a serological marker.

## Conclusions

One-third of children having short stature have celiac disease. The frequency of celiac disease in children with short stature has no association with gender and age. Celiac disease was more common in children whose parents were either illiterate or had a primary level of education. No statistically significant association was found between family type and celiac disease. 
